# Case Report: Recurrent cerebral embolism secondary to direct cardiac invasion by lung adenocarcinoma

**DOI:** 10.3389/fcvm.2026.1688578

**Published:** 2026-06-24

**Authors:** Xiangxiong Wu, Shenwen He, Yayao Huo, Yuying Wu, Xiaoying Li, Ying Yang, Dawei Dong

**Affiliations:** 1 Department of Neurology, The Affiliated Shunde Hospital of Jinan University, Foshan, China; 2 Department of Neurology and Stroke Center, The First Affiliated Hospital of Jinan University, Guangzhou, China

**Keywords:** cardioembolic stroke, lung adenocarcinoma, neurocardiology, recurrent cerebral embolism, tumor invasion of heart

## Abstract

**Background:**

Recurrent cerebral embolism due to direct cardiac invasion by lung cancer is exceedingly rare yet clinically significant, representing a convergence of neuro-oncologic and cardiogenic stroke mechanisms.

**Case description:**

A 41-year-old woman presented with acute right-sided hemiparesis and aphasia. Imaging revealed left middle cerebral artery occlusion, treated successfully with mechanical thrombectomy. Four days later, she developed a second embolic stroke in the right middle cerebral artery (MCA) territory—again resolved via thrombectomy. Further evaluation uncovered a right lung adenocarcinoma infiltrating the pericardium, pulmonary vein, and left atrium. The patient received systemic chemotherapy and anticoagulation. No subsequent embolic events occurred during follow-up.

**Conclusion:**

This case demonstrates a rare but important cause of recurrent cerebral embolism—direct cardiac invasion by malignancy—and highlights the efficacy of thrombectomy in cancer-associated large-vessel occlusion. Clinicians should maintain high suspicion for tumor-related stroke in atypical embolic presentations, especially in younger individuals without conventional risk factors.

## 1 Introduction

This report details a rare case of lung adenocarcinoma with direct invasion into the left atrium, causing recurrent cerebral embolic events. This case emphasizes the importance of recognizing tumor-related cerebral embolism in patients without traditional cardiovascular risk factors. In addition, in this case, vessel recanalization was successfully achieved via mechanical thrombectomy.

## 2 Case presentation

**Figure 1:**
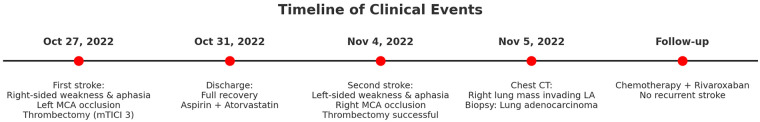
Timeline of clinical events. The patient initially presented on October 27, 2022 with right-sided weakness and aphasia caused by left middle cerebral artery (MCA) occlusion, successfully treated with mechanical thrombectomy. She was discharged on October 31, 2022 after full neurological recovery with secondary prevention therapy (aspirin and atorvastatin). On November 4, 2022, she developed a second embolic stroke due to right MCA occlusion, again treated with thrombectomy. Subsequent chest CT on November 5, 2022 revealed a right lung mass infiltrating the pericardium, pulmonary vein, and left atrium; biopsy confirmed lung adenocarcinoma. She was placed on chemotherapy and long-term anticoagulation (rivaroxaban), with no recurrence during follow-up.

A timeline of the entire clinical events is shown in Figure 1.

### 2.1 First episode (October 27, 2022)

**Figure 2:**
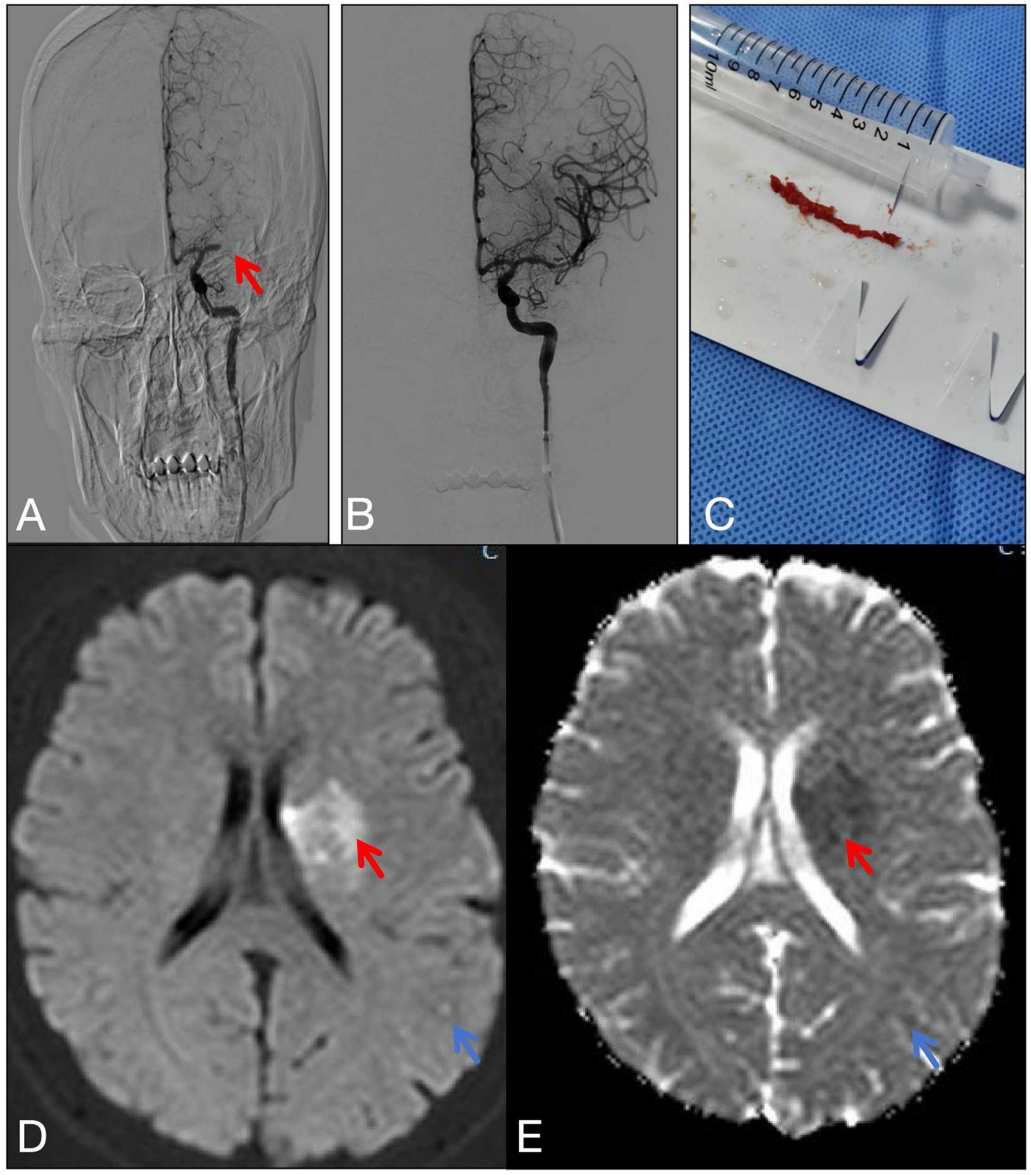
Angiography showing **(A)** occlusion of the M1 segment of the left middle cerebral artery (red arrow) and **(B)** successful revascularization. **(C)** Specimens obtained by mechanical thrombectomy. **(D)** Brain MRI-DWI showed restricted diffusion in the left basal ganglia and parieto-occipital cortex (blue arrow), and **(E)** MRI-ADC showed low signal intensity (red arrow and blue arrow). DWI = Diffusion weighted imaging; ADC = Apparent diffusion coefficient.

A 41-year-old female presented to the emergency department with sudden right-sided weakness, slurred speech, and fatigue for one hour. Upon examination, her vitals were within normal limits, but neurological assessment revealed right-sided hemiparesis (muscle strength 2/5), and an National Health Institutes of Health Stroke Scale (NIHSS) score of 11. Non-contrast brain Computed Tomography (CT) was unremarkable, but cerebral CT angiogram (CTA) revealed occlusion of the left middle cerebral artery (MCA) M1 segment. With no contraindications for thrombolysis, she was diagnosed with acute ischemic stroke due to large-vessel occlusion, prompting intravenous thrombolysis and endovascular therapy. Cerebral Digital Subtraction Angiography (DSA) confirmed the occlusion **(**Figure 2A**)**, and thrombectomy was performed using the Solitaire FR with intracranial support catheter for mechanical thrombectomy (SWIM) technique (mechanical stent retrieval and catheter aspiration), a 4 cm red clot was extracted **(**Figure 2C**)**, which resulting in complete recanalization (mTICI 3) **(**Figure 2B**)**. Her medical history was otherwise unremarkable, with no history of hypertension, diabetes, coronary artery disease, smoking, or alcohol use. Family history was non-contributory.

### 2.2 Laboratory and imaging findings

Postoperative Magnetic Resonance Imaging (MRI) revealed acute infarction in the left basal ganglia and parieto-occipital cortex **(**Figures 2D,E**).** Bedside transthoracic echocardiography (TTE) showed no significant cardiac or carotid abnormalities. Notable findings included a hepatic nodule suggestive of hemangioma and a uterine wall defect likely related to previous cesarean delivery. Electrocardiogram (ECG) and 24-hour Holter showed normal ECG with rare atrial and ventricular ectopics. Transcranial Doppler (TCD) monitoring post-surgery showed symmetric MCA blood flow without microembolic signals; A bubble test was negative, ruling out a cardiac shunt.

Laboratory tests indicated mildly low hemoglobin (109 g/L) and elevated D-dimer (1.86 mg/L).Other parameters, including liver and renal function, lipid profile, HIV, TPPA, HCY, HbA1c, and autoimmune markers, were within normal limits.

### 2.3 Outcome

Her right-sided weakness and speech improved fully following thrombectomy. Secondary prevention with aspirin and atorvastatin was initiated. Despite the favorable recovery, the etiology of her stroke remained unclear. Due to family concerns, she declined further investigation and was discharged on October 31, 2022.

### 2.4 Recurrence (November 4, 2022)

**Figure 3:**
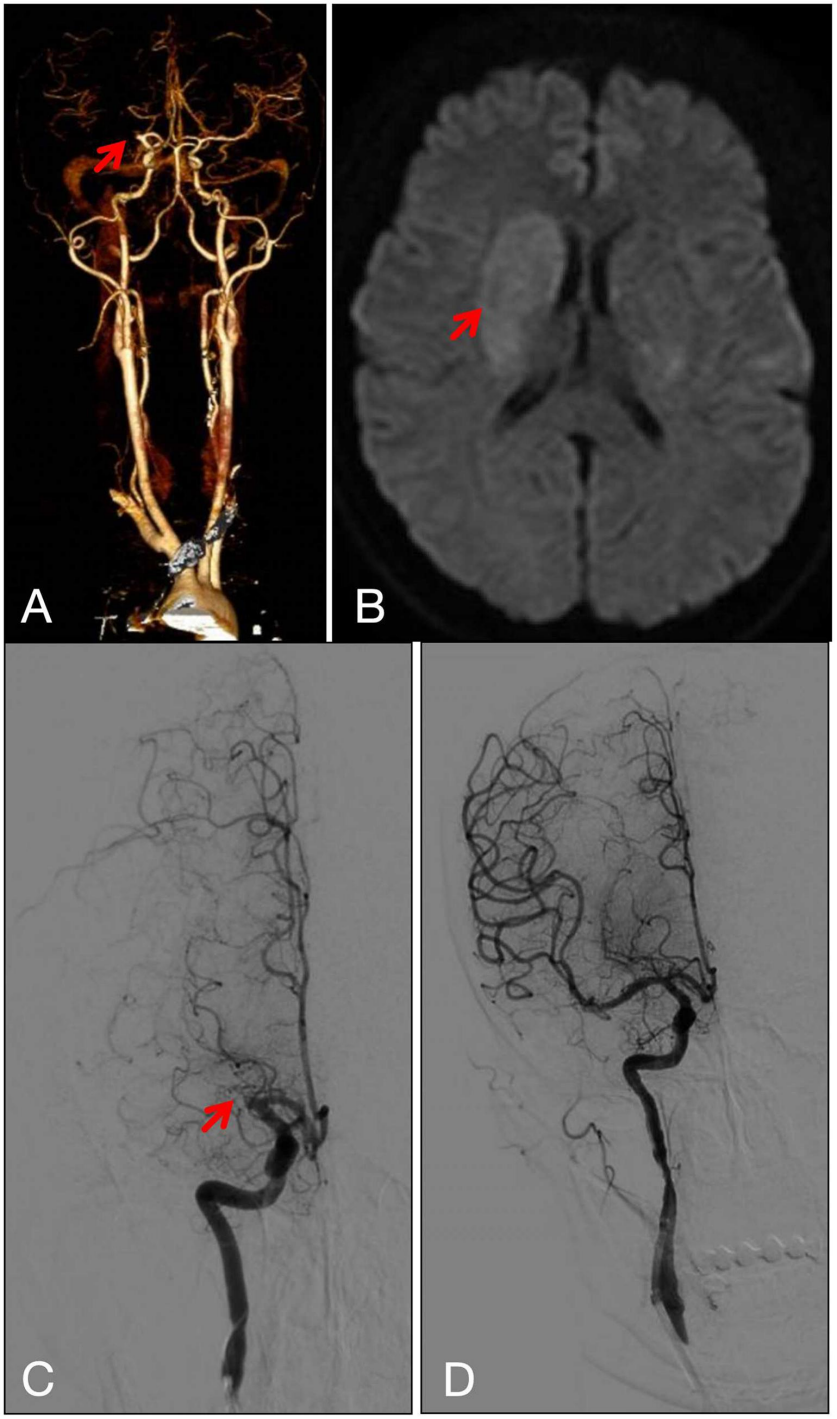
**(A)** CTA showing occlusion of the right MCA M1 segment (red arrow), **(B)** MRI-DWI showing acute infarction in the right basal ganglia (red arrow). Angiography showing **(C)** occlusion of the M1 segment of the left middle cerebral artery (red arrow) and **(D)** successful revascularization.

Four days post-discharge, she experienced sudden left-sided weakness and slurred speech. NIHSS score was 13. Repeat CTA and Diffusion-Weighted Imaging (DWI) MRI showed occlusion of the right MCA M1 segment with acute infarction in the right basal ganglia **(**Figures 3A,B**).** As the ischemic cerebral infarction had occurred four days earlier, repeat thrombolysis was not recommended because of the risk of cerebral hemorrhage. Consequently, a second thrombectomy was performed, achieving full recanalization **(**Figures 3C,D**).**

### 2.5 Further findings

**Figure 4:**
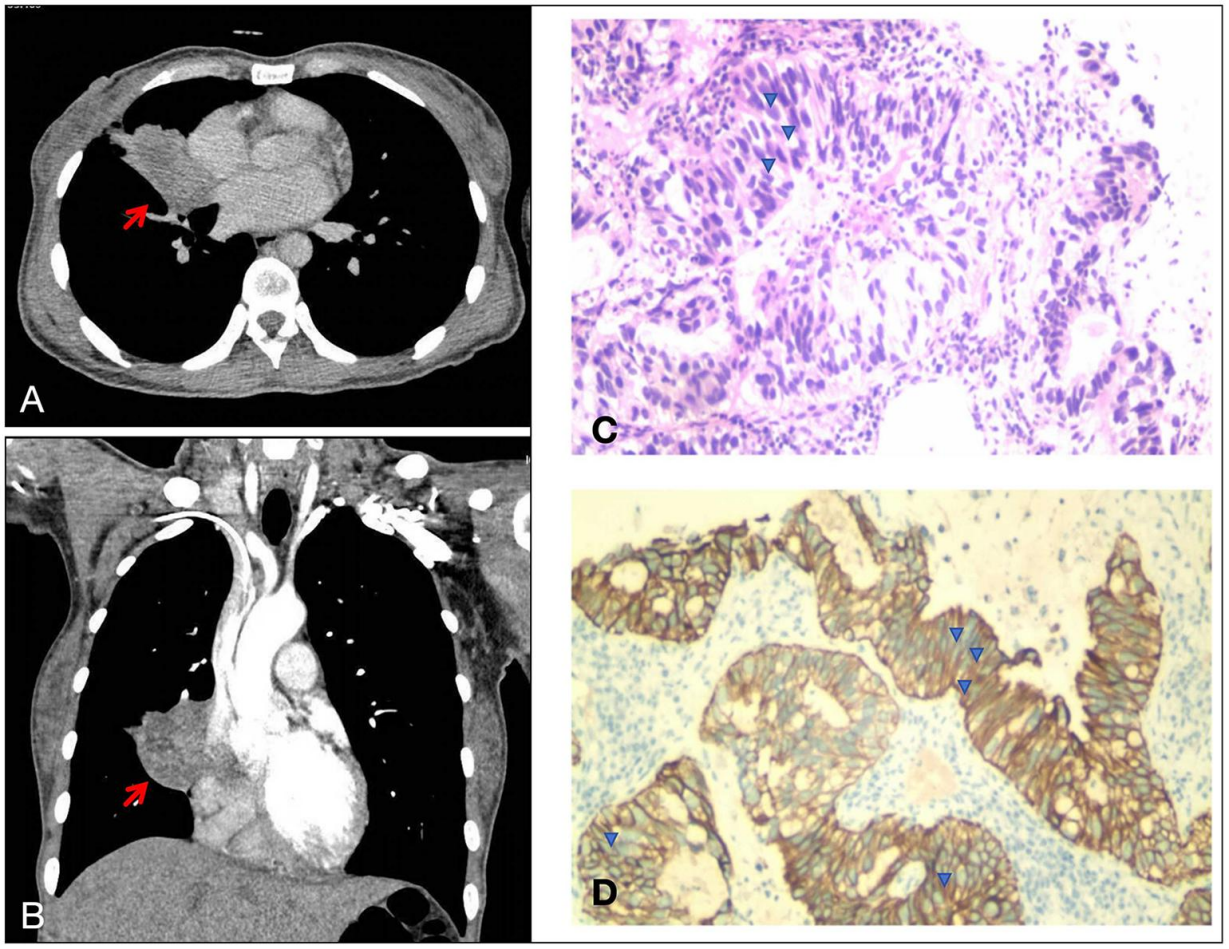
**(A,B)** CT angiogram of the chest showing a lung mass invading the pericardium, pulmonary vein, and left atrium (arrows). Representative micrographs (20  ×  ) of population of neoplastic cells via hematoxylin and eosin stain **(C)** and immunohistochemistry stain **(D)** were confirmed to be consistent with lung adenocarcinoma (blue arrows).

Postoperative chest CT revealed a 7 × 5 × 6 cm right lung mass invading the pericardium, pulmonary vein, and left atrium **(**Figures 4A,B**)**. A biopsy confirmed lung adenocarcinoma **(**Figures 4C,D**)**. Given the high surgical risk, the patient opted for conservative treatment with chemotherapy and was started on long-term anticoagulation with rivaroxaban. As of the latest follow-up (three months after the second thrombectomy), she remains free of further strokes and without neurological sequelae.

## 3 Discussion

Cerebral embolism, or embolic stroke, results from the sudden obstruction of cerebral arteries by emboli, leading to ischemia and necrosis of brain tissue. This condition has a high mortality rate, particularly in large-vessel occlusions. Cerebral embolism primarily originates from arterial-to-arterial or cardioembolic sources, with the latter accounting for 20%–30% of ischemic strokes (1). Cardioembolic sources include atrial fibrillation, cardiac mural thrombi, endocarditis vegetations, cardiac tumors, and paradoxical emboli through right-to-left shunts (2, 3).

This patient's lack of traditional risk factors for atherosclerosis pointed to an embolic origin. Following conventional diagnostic workup, common sources were ruled out. Enhanced chest CT and pathology confirmed central lung adenocarcinoma with invasion into the left atrium, suggesting a link between recurrent cerebral embolism and the malignancy. This case aligns with a previously reported case in *Neurology* (2019), where a lung squamous cell carcinoma metastasis caused direct tumor embolism in the left MCA. Literature review reveals few cases of acute stroke due to direct cardiac invasion by tumors (4). In our case, the patient's recurrent embolism within a week, affecting both hemispheres, and absence of other embolic sources indicate a probable link between the lung tumor's left atrial invasion and embolic strokes.

Embolic strokes generally have more complex etiologies, more severe symptoms, and worse prognosis than ischemic strokes due to intracranial atherosclerosis (ICAS) (5). In acute ischemic stroke, intravenous thrombolysis is the first-line therapy. Although malignancy is not an exclusion criterion, there is limited evidence for its efficacy in tumor-related stroke. Retrospective studies offer conflicting findings: some report no increase in hemorrhage or mortality in cancer patients treated with thrombolysis (6), while others show a higher risk of intracranial hemorrhage (7).

At present, most large Randomized controlled trial (RCT) studies on the endovascular treatment of acute ischemic stroke exclude patients with malignant tumors, so the selection of mechanical thrombectomy for malignant tumors require careful decision-making. In this case, initial intravenous thrombolysis followed by mechanical thrombectomy was successful, and the second stroke was resolved with thrombectomy alone, suggesting its safety and potential efficacy.

Cancer-related stroke often warrants anticoagulation due to a hypercoagulable state associated with malignancy. For cancer-related stroke, long-term anticoagulation appears reasonable. The TEACH trial showed no significant difference in outcomes between aspirin and enoxaparin in cancer-associated stroke (8). Although LMWH (Low molecular weight heparin) is typically preferred, patient adherence is higher with novel oral anticoagulants (NOACs), which have demonstrated comparable safety and efficacy (9). In this case, transitioning to rivaroxaban after lung cancer diagnosis has so far prevented recurrence.

One concern in this case is that initial bedside transthoracic echocardiography (TTE) failed to detect cardiac invasion. We consider the reason is that TTE is sufficiently limited in detecting left atrial tumors: low sensitivity due to limited acoustic windows due to lung gas and chest wall interference make it difficult to show lesions that do not clearly project into the atrial cavity (10). Moreover, repeated TTE failed to detect a large left atrial myxoma in a previously reported case, which was subsequently identified by transesophageal echocardiography (TEE) and cardiac CT (11). In our patient, enhanced CT showed tumor invasion confined to the left atrial ostium without intraluminal projections, a pattern below the TTE detection threshold. When cardiac invasion is clinically suspected and TTE is negative, contrast-enhanced CT or TEE should be pursued.

### 3.1 Implications and limitations of this case

A major limitation is the lack of histopathological examination of the retrieved thrombus (not routinely performed during emergency thrombectomy in our center), preventing confirmation of tumor cells in the clot. Based on the direct continuity between the intra-cardiac tumor mass (lung cancer invading the left atrium) and the systemic circulation, had such analysis been performed, two findings were possible: tumor cell aggregates (due to direct cardiac invasion, as supported by previous reports of malignant cells in cerebral thrombi) or bland thrombus (due to the cardiac neoplasm causing local hemodynamic disorder, it subsequently leads to blood stasis in the left atrium). Nonetheless, irrespective of the exact mechanism, the repeated embolic events in close temporal and anatomical association with lung cancer invasion of the left atrium strongly suggest a causal relationship.

### 3.2 Knowledge gaps

Despite the illustrative nature of this case, several knowledge gaps remain. First, the exact pathophysiological mechanism of recurrent cerebral embolism in patients with direct cardiac invasion by lung cancer is not fully understood. It remains unclear whether tumor cell aggregates directly detach into the circulation or whether local hemodynamic disturbance (e.g., blood stasis within the left atrium) promotes bland thrombus formation–or both. Second, the true incidence of this phenomenon is unknown, as most cases are likely underrecognized or misdiagnosed as cryptogenic stroke.Third, the optimal secondary prevention strategy – antiplatelet therapy, anticoagulation, or chemotherapy alone – has not been defined for this unique scenario.

### 3.3 Future directions

To address these gaps, future research should prioritize the following directions: (1) Multicenter case registries and collaborative networks are needed to systematically collect data on patients with tumor-related cerebral embolism due to direct cardiac invasion, which would help estimate the true incidence and describe clinical phenotypes. (2) Routine histopathological examination of retrieved thrombi during mechanical thrombectomy should be encouraged in suspected cases, as it could differentiate tumor cell aggregates from bland thrombus and provide direct evidence of the embolic source. (3) Randomized controlled trials or large observational studies are warranted to compare antiplatelet therapy, direct oral anticoagulants, and low-molecular-weight heparin for secondary stroke prevention in this population.

## 4 Patient perspective

The patient expressed that the sudden onset of two consecutive strokes within such a short period was an overwhelming and frightening experience, particularly given her young age and absence of prior health issues. She was grateful for the rapid diagnosis and the success of mechanical thrombectomy, which allowed her to recover without permanent neurological disability. Learning about the underlying cause – lung adenocarcinoma invading the heart – was initially shocking and emotionally challenging. However, she acknowledged that the timely discovery of the cancer gave her an opportunity to receive treatment earlier than she otherwise would have. She emphasized her appreciation for the multidisciplinary care team and the close follow-up, which gave her confidence in her recovery. She also expressed hope that her case could raise awareness among clinicians and patients that stroke may sometimes be the first sign of an underlying malignancy.

## 5 Conclusion

The etiology of acute ischemic stroke is complex, with cancer-related stroke as an essential subtype. Mechanisms include hypercoagulability, hemodynamic disorder, nonbacterial thrombotic endocarditis, radiation-induced vasculopathy, chemotherapy-induced vasculitis, direct tumor embolism, and vascular compression by the tumor (12, 13). This unique case involves tumor invasion directly affecting the heart and causing embolism. Early identification of stroke etiology, especially for large-vessel occlusion, is critical to prevent recurrence and improve prognosis.

## Data Availability

The original contributions presented in the study are included in the article/Supplementary Material, further inquiries can be directed to the corresponding authors.
